# A review of the current knowledge on *Zeugodacus cucurbitae* (Coquillett) (Diptera, Tephritidae) in Africa, with a list of species included in *Zeugodacus*

**DOI:** 10.3897/zookeys.540.9672

**Published:** 2015-11-26

**Authors:** Marc De Meyer, Hélène Delatte, Maulid Mwatawala, Serge Quilici, Jean-François Vayssières, Massimiliano Virgilio

**Affiliations:** 1 Royal Museum for Central Africa, Invertebrates Unit, Leuvensesteenweg 13, B3080 Tervuren, Belgium; 2 CIRAD, UMR PVBMT, 7, ch de l’IRAT, 97410 Saint-Pierre, La Réunion, France; 3 Sokoine University of Agriculture, Dept of Crop Science and Production, Morogoro, Tanzania; 4 CIRAD, UPR HortSys, 34398, Montpellier, France and IITA, Biocontrol Unit for Africa, 08 BP 0932, Cotonou, Bénin; 5 Royal Museum for Central Africa, JEMU, Leuvensesteenweg 13, B3080 Tervuren, Belgium

**Keywords:** Melon fly, Cucurbitaceae, Afrotropical, pest species

## Abstract

This paper reviews all available information regarding the occurrence and biology of the melon fly, *Zeugodacus cucurbitae* (Coquillett), in the Afrotropical Region, including data on invasion history, distribution patterns, population genetics, host range, and interspecific competition. Although limited intraspecific variability has been observed within the region regarding the above mentioned aspects, there seems to be no indication that *Zeugodacus cucurbitae* represents a species complex. A checklist of all of the species included in *Zeugodacus* as recently proposed by Virgilio et al. (2015) is provided.

## Introduction

The melon fly, *Zeugodacus cucurbitae* (Coquillett) is a major agricultural pest of Asian origin. Despite the vernacular English name and the species-group name, it is reported from a series of unrelated host families in addition to the vast host range within Cucurbitaceae (White and Elson-Harris 1994). The fact that a number of populations of *Zeugodacus cucurbitae* differ in their reported host plants, morphology, etc. from region to region, resulted in the species being included in the Coordinated Research Project on “Resolution of cryptic species complexes of tephritid pests to overcome constraints to SIT application and international trade”, initiated by the Joint FAO/IAEA Programme in 2010. This paper reviews the taxonomic position and history of the species within the Tephritidae, provides information on its worldwide distribution and genetic diversity, summarizes the current knowledge regarding the species in Africa, and provides a checklist of all of the species included in *Zeugodacus* as recently proposed by Virgilio et al. (2015).

## Classification and taxonomic history

**Figure 1.**
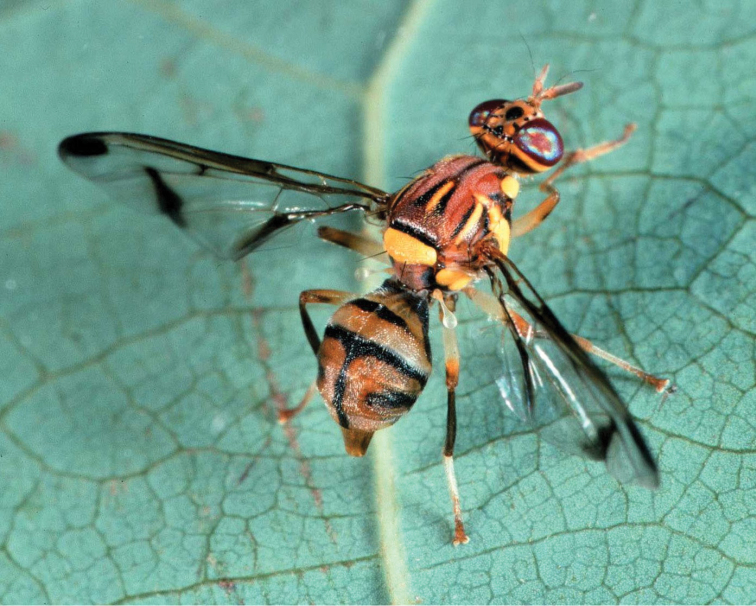
Habitus image of *Zeugodacus cucurbitae* (photo R.S. Copeland).

*Zeugodacus cucurbitae* (Figure 1) was originally described as *Dacus cucurbitae* by Coquillett (1899) from two males and two females bred from larvae found in green cucumbers in Honolulu, Hawaii (USA). *Bactrocera* was considered a subgenus of *Dacus* until Drew (1989) proposed a classification recognizing both taxa as genera, based upon the abdominal tergites being fused, (in *Dacus*), or not (in *Bactrocera*). Drew placed *Zeugodacus cucurbitae* in the subgenus *Zeugodacus*, first under *Dacus* following previous authors (Drew 1973), and later under *Bactrocera* (Drew 1989). The subgenus *Zeugodacus* belongs to a group of subgenera, characterized by the posterior lobe of the male lateral surstylus being long and the male abdominal sternite 5 being slightly concave along the posterior margin (rather than having a deep V shaped indentation) (Drew and Hancock 1999). At least 50% of the species included in the *Zeugodacus* group, for which host plant records are available, are cucurbit feeders. Recently the systematic position of *Zeugodacus* was revised as *Bactrocera*, *Dacus* and the subgenera of the *Zeugodacus* group have different evolutionary histories (Krosch et al. 2012, Virgilio et al. 2015). The molecular data provided support the hypothesis of White (2006) who suggested a common ancestry for *Zeugodacus* and *Dacus* (but see Hancock and Drew 2015 for a different hypothesis). Here we refer to the classification proposed by Virgilio et al. (2015) by using the new generic combination Zeugodacus (Zeugodacus) cucurbitae for the melon fly, although most existing literature refer to it under the former combination, Bactrocera (Zeugodacus) cucurbitae.

The genus *Zeugodacus* currently includes 192 species (see list in Supplementary material 1). Most species within this genus are restricted to the Oriental and Australasian Regions, with a few species reaching into the eastern Palearctic in China and Japan, except for *Zeugodacus cucurbitae* which was introduced into other parts of the world. *Zeugodacus cucurbitae* is rather distinctive in adult morphology and can be differentiated from other related species by the following combination of characters: scutum red-brown, with medial and lateral yellow postsutural vittae; large apical spot on the wing with posterior margin reaching about halfway between vein R_4+5_ and vein M; infuscation present over crossvein dm-cu and usually also crossvein r-m, wing cells bc and c hyaline, abdomen with a narrow transverse black band across basal margin of tergite 3 and a medial longitudinal black stripe over tergites 3-5 (White 2006, Drew and Romig 2013).

Contrary to other species like the *Bactrocera dorsalis* (Hendel) populations found in Africa (see Drew et al. 2005, White 2006), there is little intraspecific variability observed in adult *Zeugodacus cucurbitae* specimens with regard to scutal and abdominal patterns. Drew and Romig (2013) only mention that the fuscous marking on the scutum can be absent or present. White (2006) indicates that the anterior supra-alar and prescutellar acrostical setae can be rarely absent (the latter being one of the main differentiating characters between *Dacus* and most *Bactrocera* species), while the basal scutellar seta can be rarely present (hence, four setae in total rather than the usual two which are situated apically on the scutellum). The crossband on r-m is not always distinct. However, these differences do not seem to reflect any particular pattern linked to cryptic speciation but rather phenotypic plasticity. *Zeugodacus cucurbitae* was not included in the list of the Asian species complexes defined by Drew and Romig (2013). No key is available to differentiate it from all other *Zeugodacus* species. Drew (1989) provides a general key for *Bactrocera* of the Australasian and Oceanian regions, including *Zeugodacus cucurbitae* and 19 other *Zeugodacus* species, while Drew and Romig (2013) provide descriptions and some diagnostic features for 101 species from South-East Asia, but no key. White (2006) and Virgilio et al. (2014) provide a key for African Dacina including *Zeugodacus cucurbitae*.

**Figure 2.**
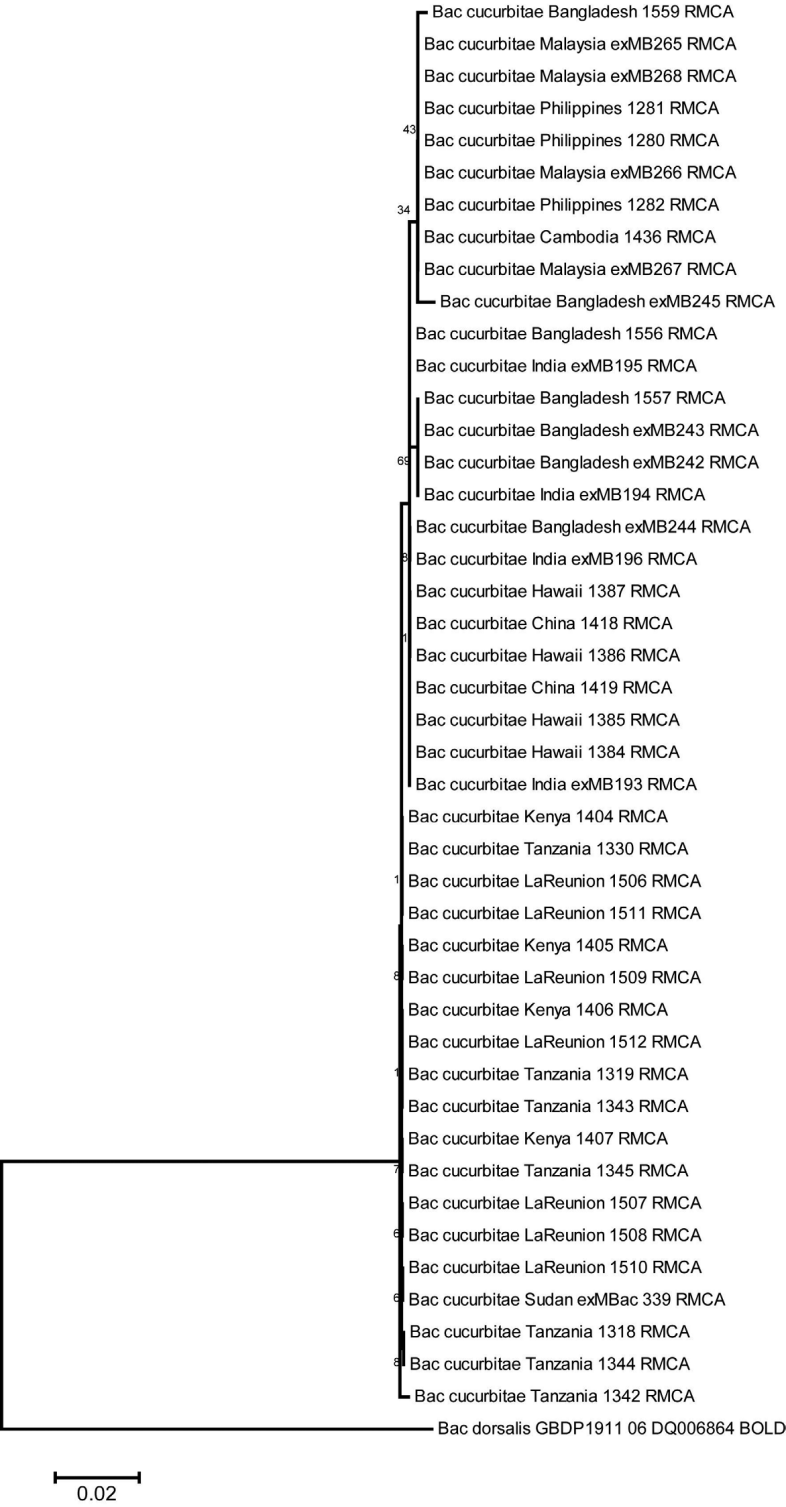
NJ tree (K2P distance, Kimura 1980) including 44 COI DNA barcodes of *Zeugodacus cucurbitae* from 11 countries (Virgilio and De Meyer, unpublished data).

DNA barcoding shows remarkably low intraspecific variability. A pilot study including COI barcodes of 44 specimens originating from 11 countries along the entire distribution range (Virgilio and De Meyer, unpublished data) revealed an average K2P genetic distance (Kimura 1980) of only 0.02% (Figure 2). Similarly, the concatenation of mitochondrial DNA sequences (COI and ND6 gene fragments) from 100 specimens from Asia, Hawaii, African continent and islands of the Indian Ocean resulted in 22 haplotypes with 21 polymorphic sites and an average p-distance of only 0.003% (Jacquard et al. 2013). Minimum Spanning Network indicated the occurrence of two main haplotype groups corresponding to specimens from (a) Asia and Hawaii, and (b) the African continent including also Reunion Island.

## Distribution, origin and population structure

Although *Zeugodacus cucurbitae* was originally described from the Hawaiian Islands, its presence there was the result of accidental human-mediated introduction (Bess 1961). About a decade later the first record from India was published (Froggatt 1909). Since then, it has been reported from multiple countries in the Asian and Australian-Oceanian Regions (Dhillon et al. 2005, Drew 1982, 1989, Drew and Romig 2013). It is abundant throughout Central and East Asia (including Pakistan, India, Bangladesh, Nepal, China, Indonesia and the Philippines) and Oceania (including New Guinea and the Mariana Islands). In some of these regions, it has been the subject of a number of introductions, eradication attempts and subsequent re-introductions. This is in particular the case in parts of the Pacific like the Northern Mariana Islands and Nauru (Dhillon et al. 2005), although it has also been successfully eradicated (Suckling et al. 2014) from regions in which it was well established, such as southern Japan in the 1990ies, using Sterile Insect Technique (Koyama et al. 2004). Since 1956 *Zeugodacus cucurbitae* has been detected a number of times in California (Papadopoulos et al. 2013), but its permanent establishment on the North American mainland is not confirmed.

In Africa, the first record dates back to 1936 from Tanzania (based upon a male specimen in the collection of the Natural History Museum in London, collected at Tanga on January 10^th^, 1936 by N. Krauss. See Bianchi and Krauss (1937) for report on this expedition, although this record is not specifically mentioned). No other species that are closely related to *Zeugodacus cucurbitae* are found in Africa, and its occurrence on the continent is also attributed to introduction. However, it is unclear whether it was introduced at that time (1936) or whether it was already present for a much longer time. There are historical ties between the eastern coastal area of Africa (dominated by the so-called Swahili culture) and the near East and Indian subcontinent that date back to 100 AD (Gilbert 2004), with movements and shipments of commodities between both regions. The first records from the African mainland were restricted to coastal Tanzania and Kenya (first record 1937) (Table 1).

**Table 1.** First records of *Zeugodacus cucurbitae* in African countries (based upon records in Orian and Moutia 1960, Vayssières et al. 2007 and De Meyer and White 2007).

Country	Locality	Year
Tanzania	Tanga	1936
Kenya	Rabai	1937
Mauritius	N/S	1942
Réunion	N/S	1972
Gambia	Brikama	1999
Ivory Coast	Korhogo	1999
Seychelles	Mahé	1999
Mali	Bamako	2000
Burkina Faso	Orodara	2000
Guinea	Foulaya	2000
Nigeria	Moruwa	2001
Cameroon	Garoua	2002
Senegal	Dakar	2003
Ghana	Sagyimase	2003
Benin	Cotonou	2004
Niger	Dosso	2004
DRCongo	Kinshasa	2006
Togo	Agou-Logopé	2006
Sudan	Singa	2006
Sierra Leone	Freetown	2006
Uganda	Jinja	2009
Burundi	Kigwena	2010
Ethiopia	Arba Minch	2010
Malawi	Kumbali	2010
Mozambique	Mocimboa da Praia	2013

**Figure 3.**
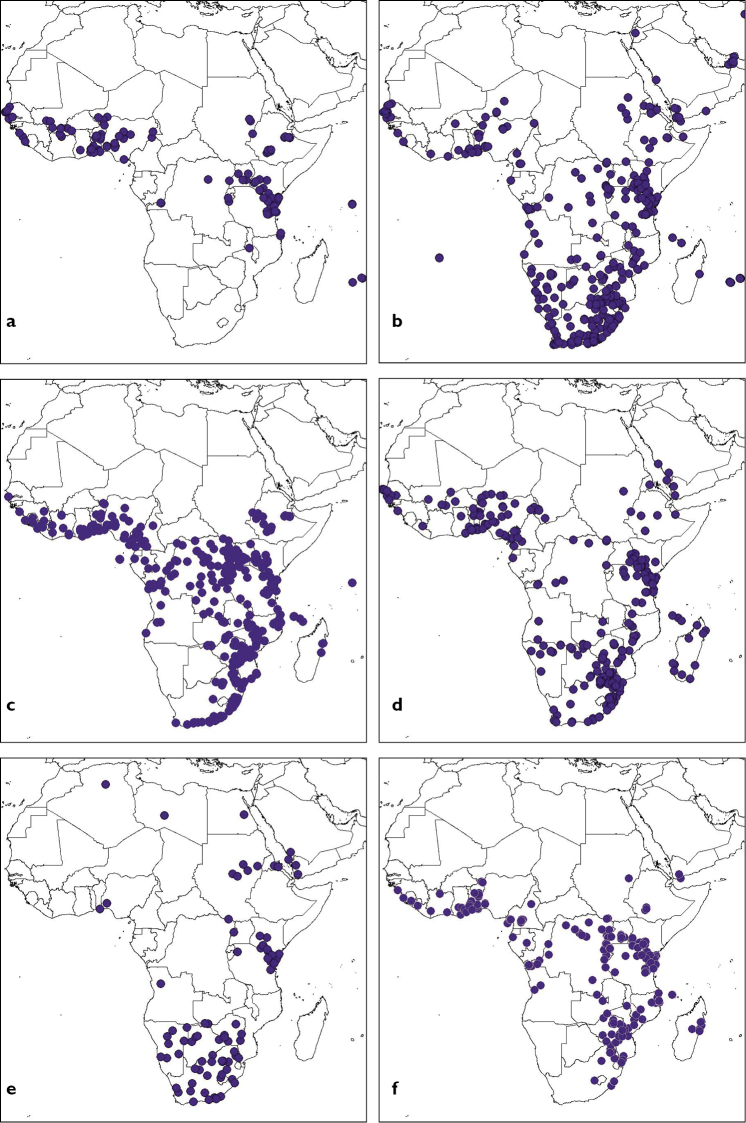
Distribution patterns for African tephritids: **a** *Zeugodacus cucurbitae* **b** *Dacus ciliatus* **c** *Dacus bivittatus* **d** *Dacus vertebratus* **e** *Dacus frontalis* **f** *Dacus punctatifrons* (source of data: http://projects.bebif.be/fruitfly/index.html).

*Zeugodacus cucurbitae* has also been introduced to several islands in the western Indian Ocean, with the first record in Mauritius in 1942 (Orian and Moutia 1960) and in La Réunion in 1972 (Vayssières 1999, White et al. 2001). More recently (since 1999) it was reported from the island Mahé of the Seychelles (White et al. 2001), where it is now also considered established. Its presence on the Comoro Archipelago is questionable (De Meyer et al. 2012) and so far no records are reported from Madagascar. Despite its longtime occurrence in eastern Africa and the Indian Ocean, *Zeugodacus cucurbitae* apparently did not spread rapidly to other parts of Africa. The first record from Central Africa was a mention in Fontem et al. (1999), where it is reported (as *Dacus cucurbitae*) as the most prevalent insect pest observed by farmers on tomatoes in Cameroon. No voucher specimens could be traced to any collections in order to confirm this record, and there is the possibility that it was based on a misidentification of another dacine attacking tomatoes. For example, *Dacus punctatifrons* Karsch has been reported as a major pest of tomato in Cameroon (Okolle and Ntonifor 2005). The first voucher specimens from West Africa that could be confirmed to belong to *Zeugodacus cucurbitae* are from Ivory Coast and the Gambia and were collected in 1999 at Korhogo and Brikama, respectively, while in 2000 one of the authors (JFV) discovered it in Mali in cuelure traps and emerging from young pumpkins. Since the beginning of the 21^st^ Century, several records of *Zeugodacus cucurbitae* from West and Central Africa became known (Table 1) and it is now well established in most parts of the region (Vayssières et al. 2007; Figure 3a).

In eastern Africa, *Zeugodacus cucurbitae* has been reported from a much larger range than just Kenya and Tanzania and it is now found from Ethiopia and the Sudan to Malawi and northern Mozambique (Table 1). It is unclear whether these 21^st^ century records are a true reflection of a further recent expansion of its geographical range or that they are due to incomplete sampling in preceding decades. However, the currently observed dispersal of this species has also increased the awareness of its economic significance. *Zeugodacus cucurbitae* has been considered a major pest species of commercially grown cucurbits in large parts of Asia (Kapoor 1989, Koyama 1989) and Hawaii (Harris 1989) for a long time. However, in the Afrotropical region, not much research was devoted to this species in comparison to other cucurbit infesting dacines, except for La Réunion (White and Elson-Harris 1994, Vayssières 1999, Ryckewaert et al. 2010) and Mauritius (Sookar et al. 2012, 2013). This is currently changing due to the recent observations on its distribution and dominance in particular crops (see below under ‘host range and interspecific competition’).

**Figure 4.**
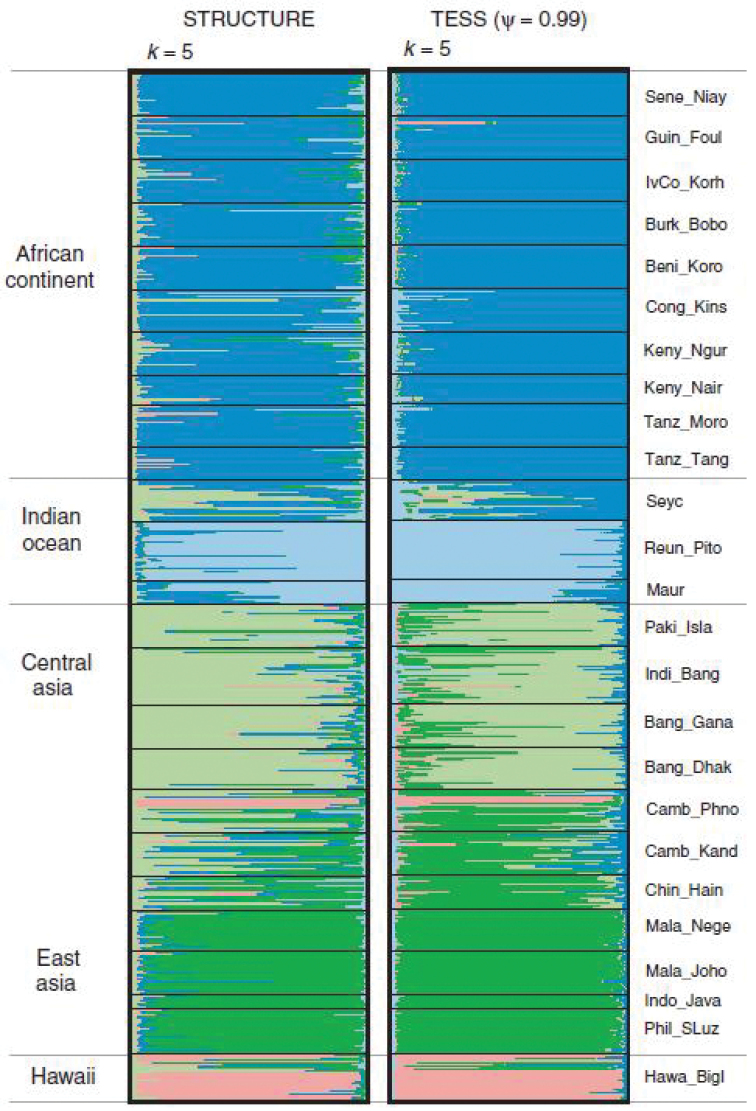
Individual admixture proportions (K=5) of 25 different populations of *Zeugodacus cucurbitae* (after Virgilio et al. 2010).

Given the current geographic distribution of other *Zeugodacus* species (all restricted to the Oriental, Australasian and eastern Palearctic Regions) and the historical data of its occurrences in Africa and Hawaii, it is generally assumed that *Zeugodacus cucurbitae* originated in the Oriental Region and that its current distribution in Africa and in other parts of the world is the result of several invasion events (see Virgilio et al. 2010). The analyses by Jacquard et al. (2013) of sequences obtained from samples from throughout the known distribution range of *Zeugodacus cucurbitae* revealed a main genetic split between samples from (a) Asia and Hawaii, and (b) Sub Saharan Africa and La Réunion Island. The main differences between the African and all other samples suggested a bottleneck(s) following introduction, yet this model was not supported by the studies of Virgilio et al. (2010). Relationships between populations from different geographic areas were further resolved through a macrogeographic population structure analysis based on 25 populations genotyped at 12 microsatellite loci (Virgilio et al. 2010). Populations could be subdivided into five main geographic groups (African continent, Western Indian Ocean islands, Indian Subcontinent, South-East Asia, and Hawaii; Fig. 4).

Levels of genetic diversity and individual Bayesian assignments (Virgilio et al. 2010) seem to suggest that *Zeugodacus cucurbitae* originated on the Indian Subcontinent and might have expanded its range to South-East Asia and Hawaii on one hand and to Africa and the Indian Ocean islands on the other (although recent anthropogenic transport might have contributed to inter-regional gene flow). Sookar et al. (2013) looked at the mating compatibility between populations of Mauritius, the Seychelles and Hawaii but only found random, non-assortative mating between the populations. Within La Réunion, Jacquard et al. (2013) also described the occurrence of local genetic clusters with distinct distributions across the eastern and western coast of the island. These clusters have possible African origin and are interconnected by high levels of gene flow both within La Réunion and between La Réunion and the African mainland.

## Host range and interspecific competition

Dhillon et al. (2005) list 81 plant species, including several non-cucurbits, as possible hosts for *Zeugodacus cucurbitae*. However, several of these hosts are considered doubtful because they were either based on casual observations (White and Elson-Harris 1994) or they are a result of induced oviposition under laboratory conditions. The latter approach provides unreliable data regarding the true natural host range of any fruit fly and should be considered with caution when determining host status (Aluja and Mangan 2008). De Meyer et al. (2007) list 45 plant species, belonging to 9 different families, that are considered hosts of *Zeugodacus cucurbitae* in Africa (including Indian Ocean islands) (Table 2).

**Table 2.** Host records for *Zeugodacus cucurbitae* from Africa.

Family	Scientific name	Country, Reference
Anacardiaceae	*Anacardium occidentale* L.	Benin, Burkina Faso: Vayssières et al. 2007
Anacardiaceae	*Mangifera indica* L.	Benin, Mali: Vayssières et al. 2008; Ivory Coast: Hala et al. 2008; Tanzania: Mwatawala et al. 2010; Mauritius: Quilici and Jeuffrault 2001
Annonaceae	*Annona senegalensis* Pers.	Western Africa: Vayssières et al. 2007
Cucurbitaceae	*Citrullus colocynthis* (L.) Schrader	Réunion: Vayssières 1999; Mauritius and Réunion: Quilici and Jeuffrault 2001
Cucurbitaceae	*Citrullus lanatus* (Thunb.) Matsum. and Nakai	Western Africa: Vayssières et al. 2007; Tanzania: Mwatawala et al. 2010; Réunion: Vayssières 1999; Mauritius and Réunion: Quilici and Jeuffrault 2001
Cucurbitaceae	*Coccinia grandis* (L.) Voigt	Kenya: White 2006; Copeland et al. 2009; Tanzania: Mwatawala et al. 2010; Réunion: Vayssières 1999; Quilici and Jeuffrault 2001
Cucurbitaceae	*Coccinia trilobata* (Cogn.) C. Jeffrey	Kenya: Copeland et al. 2009
Cucurbitaceae	*Cucumeropsis mannii* Naud.	Benin: Vayssières et al. 2007
Cucurbitaceae	*Cucumis anguria* L.	Réunion: Vayssières 1999; Quilici and Jeuffrault 2001
Cucurbitaceae	*Cucumis dipsaceus* Ehrenb. ex Spach	Kenya: White 2006; Copeland et al. 2009; Tanzania: Mwatawala et al. 2010
Cucurbitaceae	*Cucumis figarei* Naud.	Kenya: White 2006
Cucurbitaceae	*Cucumis ficifolius* A. Rich	Kenya: Copeland et al. 2009
Cucurbitaceae	*Cucumis melo* L.	Western Africa: Vayssières et al. 2007; Tanzania: Mwatawala et al. 2010; Réunion: Vayssières 1999; Mauritius and Réunion: Quilici and Jeuffrault 2001
Cucurbitaceae	*Cucumis sativus* L.	Kenya: White 2006; Copeland et al. 2009; Tanzania: White 2006; Mwatawala et al. 2010; Western Africa: Vayssières et al. 2007; Mauritius: Sookar et al. 2012; Réunion: Vayssières 1999
Cucurbitaceae	*Cucurbita maxima* Duchesne ex Lam.	Western Africa: Vayssières et al. 2007; Mauritius: Sookar et al. 2012; Réunion: Vayssières 1999
Cucurbitaceae	*Cucurbita moschata* Duchesne	Tanzania: Mwatawala et al. 2010
Cucurbitaceae	*Cucurbita pepo* L.	Western Africa: Vayssières et al. 2007; Mauritius: Sookar et al. 2012; Réunion: Vayssières 1999
Cucurbitaceae	*Cucurbita* sp.	Kenya: 1937; South African National Collections Pretoria (South Africa) data; Tanzania: Mwatawala et al. 2010
Cucurbitaceae	*Cyclanthera pedata* (L.) Schrader	Réunion: Vayssières 1999; Quilici and Jeuffrault 2001
Cucurbitaceae	*Diplocyclos palmatus* (L.) C.Jeffrey	Kenya: White 2006; Copeland et al. 2009
Cucurbitaceae	*Kedrostis leloja* (J.Gmel.) C.Jeffrey	Kenya: White 2006; Copeland et al. 2009
Cucurbitaceae	*Lagenaria leucaritha* (Dush) Pusby	Mauritius and Réunion: Quilici and Jeuffrault 2001
Cucurbitaceae	*Lagenaria sphaerica* (Sond.) Naudin	Mauritius and Réunion: Quilici and Jeuffrault 2001; Réunion: Vayssières 1999
Cucurbitaceae	*Lagenaria siceraria* (Molina) Standl.	Western Africa: Vayssières et al. 2007; Tanzania: Mwatawala et al. 2010; Réunion: Vayssières 1999
Cucurbitaceae	*Luffa acutangula* (L.) Roxb.	Tanzania: Mwatawala et al. 2010; Mauritius and Réunion: Quilici and Jeuffrault 2001; Réunion: Vayssières 1999
Cucurbitaceae	*Luffa cylindrica* M.Roem.	Western Africa: Vayssières et al. 2007; Mauritius and Réunion: Quilici and Jeuffrault 2001; Réunion: Vayssières 1999
Cucurbitaceae	*Momordica charantia* L.	Kenya: White 2006; Western Africa: Vayssières et al. 2007; Tanzania: Mwatawala et al. 2010; Mauritius and Réunion: Quilici and Jeuffrault 2001; Réunion: Vayssières 1999
Cucurbitaceae	*Momordica foetida* Schumach.	Kenya: White 2006; Copeland et al. 2009; Tanzania: Mwatawala et al. 2010
Cucurbitaceae	*Momordica rostrata* A. Zimm.	Kenya: Copeland et al. 2009; Tanzania: Mwatawala (pers.observations)
Cucurbitaceae	*Momordica trifoliata* Hook. f.	Kenya: White 2006; Copeland et al. 2009; Tanzania: Mwatawala et al. 2010
Cucurbitaceae	*Sechium edule* (Jacq.) Sw.	Mauritius and Réunion: Quilici and Jeuffrault 2001; Réunion: Vayssières 1999
Cucurbitaceae	*Trichosanthes cucumerina* L.	Mauritius and Réunion: Quilici and Jeuffrault 2001; Réunion: Vayssières 1999
Cucurbitaceae	*Telfairia occidentalis* Hook	Ivory Coast: Vayssières et al. 2007
Cannellaceae	*Warburgia ugandensis* Sprague	Kenya: Munro 1984
Caricaceae	*Carica papaya* L.	Tanzania: Mwatawala et al. 2010
Oxalidaceae	*Averrhoa carambola* L.	Benin, Ivory Coast: Vayssières et al. 2007
Passifloraceae	*Passiflora edulis* Sims	Réunion: Vayssières 1999; Quilici and Jeuffrault 2001
Rutaceae	*Citrus reticulata* Blanco	Benin: Vayssières et al. 2007
Rutaceae	*Citrus sinensis* Osbeck	Benin, Burkina Faso: Vayssières et al. 2007
Solanaceae	*Capsicum annuum* L. var. *longum* DC	Tanzania: Mwatawala et al. 2010.
Solanaceae	*Capsicum frutescens* L.	Western Africa: Vayssières et al. 2007
Solanaceae	*Solanum lycopersicum* L.	Réunion: Vayssières 1999; Tanzania: Mwatawala et al. 2010
Solanaceae	*Solanum aethiopicum* L.	Tanzania: Mwatawala et al. 2010
Solanaceae	*Solanum anguivi* Lam.	Tanzania: Mwatawala et al. 2010
Solanaceae	*Solanum macrocarpon* L.	Tanzania: Mwatawala et al. 2010
Solanaceae	*Solanum nigrum* L.	Tanzania: Mwatawala et al. 2010

The majority of these records are based on rearing of infested fruits collected in the wild. Twenty-nine of them are Cucurbitaceae. *Cucumis* spp. (in particular cucumber (*Cucumis sativus* L.) and melon (*Cucumis melo* L.)) and *Momordica* spp. (in particular Momordica cf trifoliata Hook. f. and bitter gourd (*Momordica charantia* L.)) were the preferential hosts both in West and East African studies (western Africa: Vayssières et al. 2007; Tanzania: Mwatawala et al. 2010). These studies have shown that in general cucurbit hosts are preferred over non-cucurbit hosts, with very low infestation rates and incidences in the latter. However, Vayssières et al. (2007) indicated that there are geographical differences with *Zeugodacus cucurbitae* being more oligophagous on La Réunion Island (with no genetic differences between flies infesting wild and cultivated hosts, see Jacquard et al. 2013), while having a broader host range in western Africa. Also, infestations rates can differ according to the region. For example *Cucumis melo* yielded 26-50 specimens/kg of fruits in West Africa, compared to 51–75 in Réunion, and more than 100 in Tanzania. *Lagenaria siceraria* (Molina) Standl. yielded very low numbers in West and East Africa but more than 100 specimens/kg in Réunion. These examples are, however, based on too limited a number of samples to draw definite conclusions, and it is not clear what are all of contributing causes of these differences in infestation rates. Seasonal differences (Mwatawala et al. 2009), weather variability, host availability, and interspecific competition could also be factors (Mwatawala et al. 2009, 2010, Vayssières et al. 2008). Although the low preference for non-cucurbit hosts has limited impact on actual crop loss, the mere presence in commercial hosts, such as mango (*Mangifera indica* L.), citrus (*Citrus* spp.) or carambola (*Averrhoa carambola* L.), can have regulatory implications for export of particular commodities. On the other hand, other polyphagous fruit fly species in Africa, such as *Bactrocera dorsalis*, *Ceratitis capitata* (Wiedemann) or *Ceratitis rosa* Karsch, which attack these commercial non-cucurbit hosts, are rarely encountered in Cucurbitaceae (Mwatawala et al. 2009).

**Figure 5.**
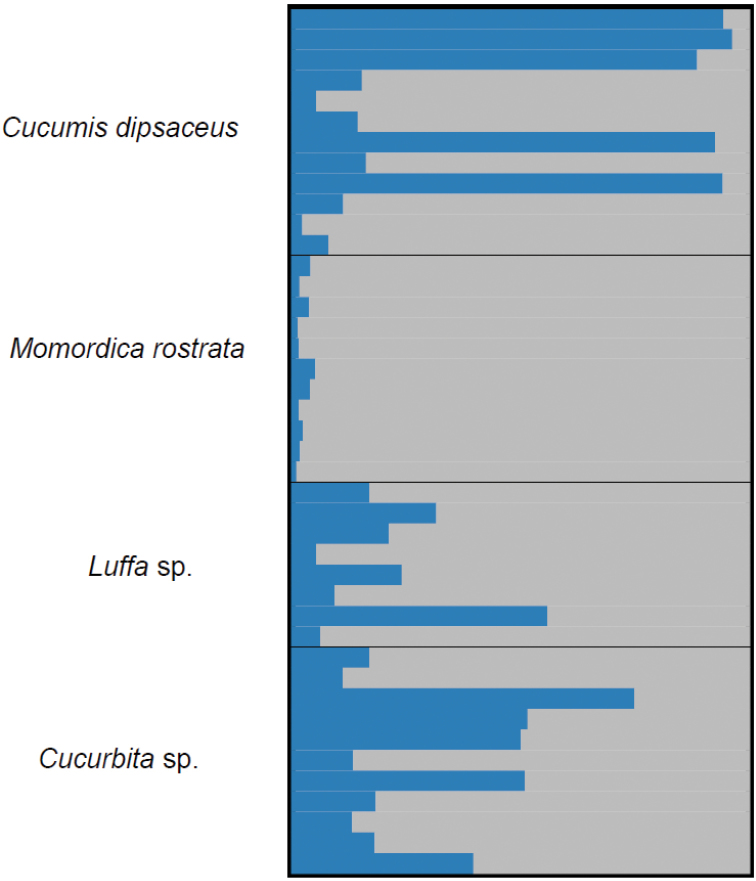
*Zeugodacus cucurbitae* specimens (n = 42) reared from four different hosts (*Cucumis dipsaceus*, *Cucurbita* sp., *Luffa* sp., *Momordica rostrata*) at the Sokoine University of Agriculture (Morogoro, Tanzania) and genotyped at 19 microsatellite loci (Mwatawala, Virgilio, De Meyer, unpublished data).

While no other *Zeugodacus* species occurs in Africa, various indigenous dacines belonging to the genus *Dacus* are known cucurbit pests, the most noteworthy and widespread being *Dacus ciliatus* Loew, *Dacus bivittatus* (Bigot), *Dacus vertebratus* Bezzi, *Dacus frontalis* Becker, and *Dacus punctatifrons*. All these species have a large geographic overlap with *Zeugodacus cucurbitae* (Figure 3b–f) and there is thus, interspecific competition for the same larval food source. Studies on the interspecific interactions between these cucurbit feeders in Africa are, however, scarce. Mwatawala et al. (2010) studied the host range and relative abundance of cucurbit feeders in central Tanzania. They concluded that *Zeugodacus cucurbitae* dominated most cucurbit hosts, in comparison to the indigenous *Dacus* species. Only *Dacus ciliatus* was predominant in some hosts like *Citrullus lanatus* (Thunb.) Matsum. and Nakai (and *Momordica charantia* to a lesser extent). A pilot study exploring genetic differentiation between 42 Tanzanian *Zeugodacus cucurbitae* specimens reared from different cucurbit hosts (*Cucumis dipsaceus* Ehrenb. ex Spach, *Cucurbita* sp., *Luffa* sp., *Momordica rostrata* A. Zimm.) and genotyped at 19 microsatellite loci did not suggest the occurrence of possible host races (Figure 5)

On La Réunion Island (1996-1999), three species (*Zeugodacus cucurbitae*, *Dacus ciliatus*, and *Dacus demmerezi* (Bezzi)) infested a range of 16 cucurbit species (Vayssières and Carel 1998; Vayssières 1999). The altitudinal limits of *Zeugodacus cucurbitae*, *Dacus ciliatus* and *Dacus demmerezi* were, respectively, 1200m, 1400m, and 1600m during the hot season. These three species have an overlap on all cucurbit crops up to 600m during the cold season and until 1200m during the hot season. At least one abiotic factor (altitude) and two biotic ones (host availability, interspecific competition) are the main screening factors for species-dominance in La Réunion. Among the 16 cucurbit hosts, *Dacus ciliatus* dominated in the cultivated hosts *Citrullus colocynthis* (L.) Schrader, *Cyclanthera pedata* (L.) Schrader, *Secchium edule* (Jacq.) Sw., and several cultivars of *Cucumis melo* and *Cucurbita pepo* L., which were cultivated above the altitudinal limit of *Zeugodacus cucurbitae* (600m during the cold season and up to 1200 meters during the hot season). *Zeugodacus cucurbitae* dominated on wild species such as *Coccinia grandis* (L.) Voigt., *Cucumis anguria* L., *Lagenaria sphaerica* (Sond.) Naudin, *Momordica charantia*, and also cultivated ones such as *Citrullus lanatus*, *Cucumis melo*, *Cucumis sativus*, *Curcubita pepo*, *Luffa acutangula* (L.) Roxb., *Luffa cylindrica* M. Roem., *Momordica* sp., and *Trichosanthes cucumerina* L. (Vayssières 1999). Vayssières et al. (2008) compared in detail the demography of *Zeugodacus cucurbitae* and *Dacus ciliatus* on La Réunion. They concluded that both species have a distinctly different life-history pattern with *Zeugodacus cucurbitae* being characterized by a later onset of reproduction, a longer oviposition time, longer life span and higher fecundity, while *Dacus ciliatus* has earlier reproduction, lower oviposition time, shorter life span and lower fecundity.

These differences in demography seem to lead to exploitative and interference competition between the two species (and most likely other cucurbit infesters as well), with *Zeugodacus cucurbitae* having an advantage over *Dacus ciliatus*. This predominance is suggested by the majority of infestations in wild cucurbit species in the field by *Zeugodacus cucurbitae*. Duyck et al. (2004) reviewed the invasion biology of (polyphagous) fruit flies and demonstrated that presence of several introduced species in areas already occupied by other tephritids, results in a decrease in number and niche shift of the pre-established species. This is largely governed by life-history strategies that species adopt for interactions in near-optimal conditions. Although the review focused on polyphagous species, a similar scenario should be considered for oligophagous pests like *Zeugodacus cucurbitae*. So far, all studies indicate that *Bactrocera* species are best adapted to exploit and to compete with other species in the same ecological niche (Duyck et al. 2006, Vayssières et al. 2008). It has also been suggested that host-range can allow niche differentiation (Duyck et al. 2008) and that this could be the reason for the different host ranges observed for *Zeugodacus cucurbitae* in La Réunion versus West Africa (Vayssières et al. 2007), with *Zeugodacus cucurbitae* being more polyphagous in West Africa. While only two indigenous cucurbit-feeding fruit flies are found on La Réunion (Vayssières and Carel 1998, De Meyer et al. 2012), at least nine are reported from West Africa (De Meyer et al. 2013). This could reflect higher interspecific competition in the latter case, with occasional shifts of *Zeugodacus cucurbitae* to non-cucurbits.

In addition to interspecific competition, the host availability and ecological niches will also affect the occurrence and impact of *Zeugodacus cucurbitae*. Earlier studies in Hawaii have shown that it is a species that is mainly found in warmer areas and that its abundance declines with increasing rainfall and increasing elevation (Vargas et al. 1989). This preference for warmer periods was confirmed in studies in La Réunion (Vayssières 1999). Studies in Tanzania showed that *Zeugodacus cucurbitae* was either absent or relatively less abundant at higher elevations along a transect from approx. 600 masl to 1650 masl. However, the exact relationship between these biotic and abiotic factors that can have an impact on the host range in different African populations, is currently poorly known and requires further investigation.

## Conclusion

Morphologically and genetically *Zeugodacus cucurbitae* shows mating compatibility among test populations and limited intraspecific genetic and morphological variability. It is still not clear if the relatively recent records for this species on the African mainland (1930s in East Africa, beginning of 21^st^ century in West Africa) are the result of local expansions of already established African populations or of one or more introductions from non-African sources. Regardless differences in host range reported across African populations there is no evidence supporting the existence of genetically isolated host races with specific feeding preferences and the observed host range variability seems more to be related to factors such as interspecific competition, host availability, and ecological niche partitioning. Although our study focused on the African populations, there is no indication that the situation might differ across the distribution of *Zeugodacus cucurbitae*.

## Supplementary materials

Supplementary material 1 Genus *Zeugodacus* (Diptera, Tephritidae), list of valid species Data type: list of species Explanation note: This list includes species listed under subgenera *Asiadacus*; *Austrodacus*; *Diplodacus*; *Hemigymnodacus*, comb. n.; *Heminotodacus*; *Hemiparatridacus*; *Nesodacus*; *Niuginidacus*; *Papuodacus*; *Paradacus*; *Parasinodacus*, comb. n.; *Sinodacus*; and *Zeugodacus*. This dataset is made available under the Open Database License (http://opendatacommons.org/licenses/odbl/1.0/). The Open Database License (ODbL) is a license agreement intended to allow users to freely share, modify, and use this Dataset while maintaining this same freedom for others, provided that the original source and author(s) are credited.Marc De Meyer, Hélène Delatte, Maulid Mwatawala, Serge Quilici, Jean-François Vayssières, Massimiliano Virgilio
