# Supplementary material for: A review of the current knowledge on Zeugodacus cucurbitae (Coquillett) (Diptera, Tephritidae) in Africa, with a list of species included in Zeugodacus
Source: Zookeys. 2015 Nov 26;(540):539–57. doi: 10.3897/zookeys.540.9672 (PMC4714087; doi:10.3897/zookeys.540.9672)
Supplement: Supplementary material 1 — Genus Zeugodacus (Diptera, Tephritidae), list of valid species [file zookeys-540-539-s001.docx]

**Genus *Zeugodacus* (Diptera, Tephritidae), list of valid species.**

**This list includes species listed under subgenera *Asiadacus; Austrodacus; Diplodacus; Hemigymnodacus*, comb. nov.; *Heminotodacus; Hemiparatridacus; Nesodacus; Niuginidacus; Papuodacus; Paradacus; Parasinodacus*, comb. nov.*; Sinodacus;* and *Zeugodacus.***

**Note: we hereby follow the recent changes and redefinitions given by Hancock and Drew (2015) except for inclusion of subgenera *Javadacus* (see Virgilio et al. 2015) and *Aglaodacus* (one species, *B. nesiotes*, where we follow White 2006 and leave it under *Daculus*).**

1. *Zeugodacus* (*Asiadacus*) *absolutus* (Walker)*,* comb. nov.
2. *Zeugodacus* (*Asiadacus*) *apicalis* (Meijere)*,* comb. nov.
3. *Zeugodacus* (*Asiadacus*) *apiciflavus* (Yu, He & Chen)*,* comb. nov.
4. *Zeugodacus* (*Asiadacus*) *atypicus* (White & Evenhuis)*,* comb. nov.
5. *Zeugodacus* (*Asiadacus*) *bakeri* (Bezzi)*,* comb. nov.
6. *Zeugodacus* (*Asiadacus*) *careomacula* (Drew & Romig)*,* comb. nov.
7. *Zeugodacus* (*Asiadacus*) *maculifacies* (Hardy)*,* comb. nov.
8. *Zeugodacus* (*Asiadacus*) *melanopsis* (Hardy)*,* comb. nov.
9. *Zeugodacus* (*Austrodacus*) *alampetus* (Drew)*,* comb. nov.
10. *Zeugodacus* (*Austrodacus*) *atrisetosus* Perkins*,* stat. rev.
11. *Zeugodacus* (*Austrodacus*) *cucumis* (French)*,* comb. nov.
12. *Zeugodacus* (*Austrodacus*) *unichromatus* (Drew)*,* comb. nov.
13. *Zeugodacus* (*Diplodacus*) *signatifer* (Tryon)*,* comb. nov.
14. *Zeugodacus* (*Hemigymnodacus*) *diversus* (Coquillett)*,* comb. nov.
15. *Zeugodacus* (*Hemigymnodacus*) *mukiae* (Drew & Romig)*,* comb. nov.
16. *Zeugodacus* (*Heminotodacus*) *dissidens* (Drew)*,* comb. nov.
17. *Zeugodacus* (*Hemiparatridacus*) *abdoaurantiacus* (Drew)*,* comb. nov.
18. *Zeugodacus* (*Nesodacus*) *atrichus* (Bezzi)*,* comb. nov.
19. *Zeugodacus* (*Niuginidacus*) *singularis* (Drew)*,* comb. nov.
20. *Zeugodacus* (*Papuodacus*) *complicatus* (White)*,* comb. nov.
21. *Zeugodacus* (*Papuodacus*) *fereuncinatus* (Drew & Romig)*,* comb. nov.
22. *Zeugodacus* (*Papuodacus*) *maculifemur* Hering*,* stat. rev.
23. *Zeugodacus* (*Papuodacus*) *neopallescentis* (Drew)*,* comb. nov.
24. *Zeugodacus* (*Papuodacus*) *ochrosterna* (Drew & Romig)*,* comb. nov.
25. *Zeugodacus* (*Papuodacus*) *sinensis* (Yu, Bai & Chen)*,* comb. nov.
26. *Zeugodacus* (*Paradacus*) *abdopallescens* (Drew)*,* comb. nov.
27. *Zeugodacus* (*Paradacus*) *angustifinis* (Hardy)*,* comb. nov.
28. *Zeugodacus* (*Paradacus*) *areolatus* (Walker)*,* comb. nov.
29. *Zeugodacus* (*Paradacus*) *aurantiventer* (Drew)*,* comb. nov.
30. *Zeugodacus* (*Paradacus*) *citroides* (Drew)*,* comb. nov.
31. *Zeugodacus* (*Paradacus*) *decipiens* (Drew)*,* comb. nov.
32. *Zeugodacus* (*Paradacus*) *duplicatus* (Bezzi)*,* stat. rev.
33. *Zeugodacus* (*Paradacus*) *fulvipes* (Perkins)*,* comb. nov.
34. *Zeugodacus* (*Paradacus*) *hancocki* (Drew & Romig)*,* comb. nov.
35. *Zeugodacus* (*Paradacus*) *magnicauda* (White & Evenhuis)*,* comb. nov.
36. *Zeugodacus* (*Paradacus*) *urens* (White)*,* comb. nov.
37. *Zeugodacus* (*Parasinodacus*) *ablepharus* (Bezzi)*,* comb. nov.
38. *Zeugodacus* (*Parasinodacus*) *binoyi* (Drew)*,* comb. nov.
39. *Zeugodacus* (*Parasinodacus*) *brevivitta* (Drew & Romig)*,* comb. nov.
40. *Zeugodacus* (*Parasinodacus*) *cilifer* (Hendel)*,* comb. nov.
41. *Zeugodacus* (*Parasinodacus*) *citrifuscus* (Drew & Romig)*,* comb. nov.
42. *Zeugodacus* (*Parasinodacus*) *eurylomatus* (Hardy)*,* comb. nov.
43. *Zeugodacus* (*Parasinodacus*) *incisus* (Walker)*,* comb. nov.
44. *Zeugodacus* (*Parasinodacus*) *longicaudatus* (Perkins)*,* comb. nov.
45. *Zeugodacus* (*Parasinodacus*) *pahangiae* (Drew & Romig)*,* comb. nov.
46. *Zeugodacus* (*Parasinodacus*) *pantabanganiae* (Drew & Romig)*,* comb. nov.
47. *Zeugodacus* (*Parasinodacus*) *pseudocucurbitae* (White)*,* comb. nov.
48. *Zeugodacus* (*Parasinodacus*) *vinnulus* (Hardy)*,* comb. nov.
49. *Zeugodacus* (*Parasinodacus*) *waimitaliae* (Drew & Romig)*,* comb. nov.
50. *Zeugodacus* (*Sinodacus*) *angusticostatus* (Drew)*,* comb. nov.
51. *Zeugodacus* (*Sinodacus*) *bogorensis* (Hardy)*,* comb. nov.
52. *Zeugodacus* (*Sinodacus*) *buvittatus* (Drew)*,* comb. nov.
53. *Zeugodacus* (*Sinodacus*) *disturgidus* (Yu, Deng & Chen)*,* comb. nov.
54. *Zeugodacus* (*Sinodacus*) *emarginatus* (Perkins)*,* comb. nov.
55. *Zeugodacus* (*Sinodacus*) *hamaceki* (Drew & Romig)*,* comb. nov.
56. *Zeugodacus* (*Sinodacus*) *hochii* (Zia)*,* comb. nov.
57. *Zeugodacus* (*Sinodacus*) *infestus* (Enderlein)*,* comb. nov.
58. *Zeugodacus* (*Sinodacus*) *longivittatus* (Chua & Ooi)*,* comb. nov.
59. *Zeugodacus* (*Sinodacus*) *paululus* (Drew)*,* comb. nov.
60. *Zeugodacus* (*Sinodacus*) *perpusillus* (Drew)*,* comb. nov.
61. *Zeugodacus* (*Sinodacus*) *sepikae* (Drew)*,* comb. nov.
62. *Zeugodacus* (*Sinodacus*) *speciosus* (Drew & Romig)*,* comb. nov.
63. *Zeugodacus* (*Sinodacus*) *spectabilis* (Drew & Romig)*,* comb. nov.
64. *Zeugodacus* (*Sinodacus*) *strigifinis* (Walker)*,* comb. nov.
65. *Zeugodacus* (*Sinodacus*) *surrufulus* (Drew)*,* comb. nov.
66. *Zeugodacus* (*Sinodacus*) *transversus* (Hardy)*,* comb. nov.
67. *Zeugodacus* (*Sinodacus*) *triangularis* (Drew)*,* comb. nov.
68. *Zeugodacus* (*Sinodacus*) *univittatus* (Drew)*,* comb. nov.
69. *Zeugodacus* (*Sinodacus*) *whitei* (Drew & Romig)*,* comb. nov.
70. *Zeugodacus* (*Zeugodacus*) *abdoangustus* (Drew)*,* comb. nov.
71. *Zeugodacus* (*Zeugodacus*) *abnormis* (Hardy)*,* comb. nov.
72. *Zeugodacus* (*Zeugodacus*) *aithonota* (Drew & Romig)*,* comb. nov.
73. *Zeugodacus* (*Zeugodacus*) *ambiguus* Shiraki*,* stat. rev.
74. *Zeugodacus* (*Zeugodacus*) *amoenus* (Drew)*,* comb. nov.
75. *Zeugodacus* (*Zeugodacus*) *anala* (Chen & Zhou)*,* comb. nov.
76. *Zeugodacus* (*Zeugodacus*) *anchitrichotus* (Drew)*,* comb. nov.
77. *Zeugodacus* (*Zeugodacus*) *apicofemoralis* (Drew & Romig)*,* comb. nov.
78. *Zeugodacus* (*Zeugodacus*) *armillatus* (Hering)*,* comb. nov.
79. *Zeugodacus* (*Zeugodacus*) *assamensis* (White)*,* comb. nov.
80. *Zeugodacus* (*Zeugodacus*) *atrifacies* Perkins*,* stat. rev.
81. *Zeugodacus* (*Zeugodacus*) *baliensis* (Drew & Romig)*,* comb. nov.
82. *Zeugodacus* (*Zeugodacus*) *baoshanensis* (Zhang, Ji, Yang & Chen)*,* comb. nov.
83. *Zeugodacus* (*Zeugodacus*) *bezzi*a*nus* Hering*,* stat. rev.
84. *Zeugodacus* (*Zeugodacus*) *biguttatus* (Bezzi)*,* comb. nov.
85. *Zeugodacus* (*Zeugodacus*) *borongensis* (Drew & Romig)*,* comb. nov.
86. *Zeugodacus* (*Zeugodacus*) *brachus* (Drew)*,* comb. nov.
87. *Zeugodacus* (*Zeugodacus*) *buruensis* (White)*,* comb. nov.
88. *Zeugodacus* (*Zeugodacus*) *calumniatus* (Hardy)*,* comb. nov.
89. *Zeugodacus* (*Zeugodacus*) *caudatus* (Fabricius)*,* stat. rev.
90. *Zeugodacus* (*Zeugodacus*) *choristus* May*,* stat. rev.
91. *Zeugodacus* (*Zeugodacus*) *connexus* (Hardy)*,* comb. nov.
92. *Zeugodacus* (*Zeugodacus*) *cucurbitae* (Coquillett)*,* stat. rev.
93. *Zeugodacus* (*Zeugodacus*) *curtus* (Drew)*,* comb. nov.
94. *Zeugodacus* (*Zeugodacus*) *daclaciae* (Drew & Romig)*,* comb. nov.
95. *Zeugodacus* (*Zeugodacus*) *daulus* (Drew)*,* comb. nov.
96. *Zeugodacus* (*Zeugodacus*) *depressus* Shiraki*,* stat. rev.
97. *Zeugodacus* (*Zeugodacus*) *diaphoropsis* Hering*,* stat. rev.
98. *Zeugodacus* (*Zeugodacus*) *diaphorus* (Hendel)*,* comb. nov.
99. *Zeugodacus* (*Zeugodacus*) *dorsirufus* (Drew & Romig)*,* comb. nov.
100. *Zeugodacus* (*Zeugodacus*) *dubiosus* (Hardy)*,* comb. nov.
101. *Zeugodacus* (*Zeugodacus*) *elegantulus* (Hardy)*,* comb. nov.
102. *Zeugodacus* (*Zeugodacus*) *emittens* (Walker)*,* stat. rev.
103. *Zeugodacus* (*Zeugodacus*) *exornatus* Hering*,* stat. rev.
104. *Zeugodacus* (*Zeugodacus*) *fallacis* (Drew)*,* comb. nov.
105. *Zeugodacus* (*Zeugodacus*) *flavipilosus* (Hardy)*,* comb. nov.
106. *Zeugodacus* (*Zeugodacus*) *flavolateralis* (Drew & Romig)*,* comb. nov.
107. *Zeugodacus* (*Zeugodacus*) *flavopectoralis* Hering*,* stat. rev.
108. *Zeugodacus* (*Zeugodacus*) *flavoverticalis* (Drew & Romig)*,* comb. nov.
109. *Zeugodacus* (*Zeugodacus*) *freidbergi* (White)*,* comb. nov.
110. *Zeugodacus* (*Zeugodacus*) *fulvoabdominalis* (White & Evenhuis)*,* comb. nov.
111. *Zeugodacus* (*Zeugodacus*) *fuscipennulus* (Drew & Romig)*,* comb. nov.
112. *Zeugodacus* (*Zeugodacus*) *fuscoalatus* (Drew & Romig)*,* comb. nov.
113. *Zeugodacus* (*Zeugodacus*) *gavisus* (Munro)*,* stat. rev.
114. *Zeugodacus* (*Zeugodacus*) *gracilis* (Drew)*,* comb. nov.
115. *Zeugodacus* (*Zeugodacus*) *hatyaiensis* (Drew & Romig)*,* comb. nov.
116. *Zeugodacus* (*Zeugodacus*) *havelockiae* (Drew & Romig)*,* comb. nov.
117. *Zeugodacus* (*Zeugodacus*) *heinrichi* Hering*,* stat. rev.
118. *Zeugodacus* (*Zeugodacus*) *hekouanus* (Yu, He & Yang)*,* comb. nov.
119. *Zeugodacus* (*Zeugodacus*) *hengsawadae* (Drew & Romig)*,* comb. nov.
120. *Zeugodacus* (*Zeugodacus*) *hoabinhiae* (Drew & Romig)*,* comb. nov.
121. *Zeugodacus* (*Zeugodacus*) *hodgsoniae* (Drew & Romig)*,* comb. nov.
122. *Zeugodacus* (*Zeugodacus*) *hoedi* (White)*,* comb. nov.
123. *Zeugodacus* (*Zeugodacus*) *hululangatiae* (Drew & Romig)*,* comb. nov.
124. *Zeugodacus* (*Zeugodacus*) *indentus* (Hardy)*,* comb. nov.
125. *Zeugodacus* (*Zeugodacus*) *iriomotiae* (Drew & Romig)*,* comb. nov.
126. *Zeugodacus* (*Zeugodacus*) *ishigakiensis* Shiraki*,* stat. rev.
127. *Zeugodacus* (*Zeugodacus*) *isolatus* (Hardy)*,* comb. nov.
128. *Zeugodacus* (*Zeugodacus*) *javadicus* (Mahmood)*,* comb. nov.
129. *Zeugodacus* (*Zeugodacus*) *juxtuncinatus* (Drew & Romig)*,* comb. nov.
130. *Zeugodacus* (*Zeugodacus*) *kaghanae* (Mahmood)*,* comb. nov.
131. *Zeugodacus* (*Zeugodacus*) *khaoyaiae* (Drew & Romig)*,* comb. nov.
132. *Zeugodacus* (*Zeugodacus*) *laguniensis* (Drew & Romig)*,* comb. nov.
133. *Zeugodacus* (*Zeugodacus*) *laocaiae* (Drew & Romig)*,* comb. nov.
134. *Zeugodacus* (*Zeugodacus*) *lipsanus* (Hendel)*,* stat. rev.
135. *Zeugodacus* (*Zeugodacus*) *liquidus* (Drew & Romig)*,* comb. nov.
136. *Zeugodacus* (*Zeugodacus*) *luteicinctutus* (Ito 2011)*,* comb. nov.
137. *Zeugodacus* (*Zeugodacus*) *macrophyllae* (Drew & Romig)*,* comb. nov.
138. *Zeugodacus* (*Zeugodacus*) *macrovittatus* (Drew)*,* comb. nov.
139. *Zeugodacus* (*Zeugodacus*) *maculatus* Perkins*,* stat. rev.
140. *Zeugodacus* (*Zeugodacus*) *melanofacies* (Drew & Romig)*,* comb. nov.
141. *Zeugodacus* (*Zeugodacus*) *menglanus* (Yu, Liu & Yang)*,* comb. nov.
142. *Zeugodacus* (*Zeugodacus*) *mesonotaitha* (Drew)*,* comb. nov.
143. *Zeugodacus* (*Zeugodacus*) *minimus* (Hering)*,* comb. nov.
144. *Zeugodacus* (*Zeugodacus*) *mundus* (Bezzi)*,* stat. rev.
145. *Zeugodacus* (*Zeugodacus*) *nakhonnayokiae* (Drew & Romig)*,* comb. nov.
146. *Zeugodacus* (*Zeugodacus*) *namlingiae* (Drew & Romig)*,* comb. nov.
147. *Zeugodacus* (*Zeugodacus*) *neoelegantulus* (White)*,* comb. nov.
148. *Zeugodacus* (*Zeugodacus*) *neoemittens* (Drew & Romig)*,* comb. nov.
149. *Zeugodacus* (*Zeugodacus*) *neoflavipilosus* (Drew & Romig)*,* comb. nov.
150. *Zeugodacus* (*Zeugodacus*) *neolipsanus* (Drew & Romig)*,* comb. nov.
151. *Zeugodacus* (*Zeugodacus*) *nigrifacies* Shiraki*,* stat. rev.
152. *Zeugodacus* (*Zeugodacus*) *okunii* Shiraki*,* stat. rev.
153. *Zeugodacus* (*Zeugodacus*) *pemalangiae* (Drew & Romig)*,* comb. nov.
154. *Zeugodacus* (*Zeugodacus*) *perplexus* (Walker)*,* comb. nov.
155. *Zeugodacus* (*Zeugodacus*) *persignatus* (Hering)*,* comb. nov.
156. *Zeugodacus* (*Zeugodacus*) *platamus* (Hardy)*,* comb. nov.
157. *Zeugodacus* (*Zeugodacus*) *proprescutellatus* (Zhang, Chen & Gao)*,* comb. nov.
158. *Zeugodacus* (*Zeugodacus*) *pubescens* (Bezzi)*,* comb. nov.
159. *Zeugodacus* (*Zeugodacus*) *purus* (White)*,* comb. nov.
160. *Zeugodacus* (*Zeugodacus*) *quasiinfestus* (Drew & Romig)*,* comb. nov.
161. *Zeugodacus* (*Zeugodacus*) *reflexus* (Drew)*,* comb. nov.
162. *Zeugodacus* (*Zeugodacus*) *rubellus* (Hardy)*,* comb. nov.
163. *Zeugodacus* (*Zeugodacus*) *sabahensis* (Drew & Romig)*,* comb. nov.
164. *Zeugodacus* (*Zeugodacus*) *sandaracinus* (Drew)*,* comb. nov.
165. *Zeugodacus* (*Zeugodacus*) *sasaotiae* (Drew & Romig)*,* comb. nov.
166. *Zeugodacus* (*Zeugodacus*) *scutellaris* (Bezzi)*,* stat. rev.
167. *Zeugodacus* (*Zeugodacus*) *scutellatus* (Hendel)*,* stat. rev.
168. *Zeugodacus* (*Zeugodacus*) *scutellinus* (Bezzi)*,* comb. nov.
169. *Zeugodacus* (*Zeugodacus*) *semongokensis* (Drew & Romig)*,* comb. nov.
170. *Zeugodacus* (*Zeugodacus*) *signatus* Hering*,* stat. rev.
171. *Zeugodacus* (*Zeugodacus*) *sonlaiae* (Drew & Romig)*,* comb. nov.
172. *Zeugodacus* (*Zeugodacus*) *sumbensis* Hering*,* stat. rev.
173. *Zeugodacus* (*Zeugodacus*) *synnephes* (Hendel)*,* stat. rev.
174. *Zeugodacus* (*Zeugodacus*) *tapervitta* (Mahmood)*,* comb. nov.
175. *Zeugodacus* (*Zeugodacus*) *tappanus* (Shiraki)*,* comb. nov.
176. *Zeugodacus* (*Zeugodacus*) *tau* (Walker)*,* comb. nov.
177. *Zeugodacus* (*Zeugodacus*) *tebeduiae* (Drew & Romig)*,* comb. nov.
178. *Zeugodacus* (*Zeugodacus*) *timorensis* Perkins*,* stat. rev.
179. *Zeugodacus* (*Zeugodacus*) *trichosanthes* (Drew & Romig)*,* comb. nov.
180. *Zeugodacus* (*Zeugodacus*) *trichotus* May*,* stat. rev.
181. *Zeugodacus* (*Zeugodacus*) *tricuspidatae* (Drew & Romig)*,* comb. nov.
182. *Zeugodacus* (*Zeugodacus*) *trimaculatus* (Hardy *&* Adachi)*,* comb. nov.
183. *Zeugodacus* (*Zeugodacus*) *trivandrumensis* (Drew & Romig)*,* comb. nov.
184. *Zeugodacus* (*Zeugodacus*) *ujungpandangiae* (Drew & Romig)*,* comb. nov.
185. *Zeugodacus* (*Zeugodacus*) *uncinatus* (Drew & Romig)*,* comb. nov.
186. *Zeugodacus* (*Zeugodacus*) *unilateralis* (Drew)*,* comb. nov.
187. *Zeugodacus* (*Zeugodacus*) *vargus* (Hardy)*,* comb. nov.
188. *Zeugodacus* (*Zeugodacus*) *vultus* (Hardy)*,* comb. nov.
189. *Zeugodacus* (*Zeugodacus*) *watersi* (Hardy)*,* comb. nov.
190. *Zeugodacus* (*Zeugodacus*) *yalaensis* (Drew & Romig)*,* comb. nov.
191. *Zeugodacus* (*Zeugodacus*) *yoshimotoi* (Hardy)*,* comb. nov.
192. *Zeugodacus* (*Zeugodacus*) *zahadi* (Mahmood)*,* comb. nov.
